# Sample Size Calculation of Clinical Trials Published in Two Leading Endodontic Journals 

**Published:** 2013-12-24

**Authors:** Arash Shahravan, Ali-Akbar Haghdoost, Maryam Rad, Maryamalsadat Hashemipoor, Maryam Sharifi

**Affiliations:** a *Kerman Oral and Dental Diseases Research Center, Kerman University of Medical Sciences, Kerman, Iran *; b *The Research Center for Modeling in Health, Institute for future studies in Health, Kerman University of Medical Sciences, Kerman, Iran*

**Keywords:** Clinical Trial, Endodontic, Sample Size

## Abstract

**Introduction:** The purpose of this article was to evaluate the quality of sample size calculation reports in published clinical trials in Journal of Endodontics and International Endodontic Journal in years 2000-1 and 2009-10. **Materials and Methods:** Articles fulfilling the inclusion criteria were collected. The criteria were: publication year, research design, types of control group, reporting sample size calculation, the number of participants in each group, study outcome, amount of type *I* (*α*) and *II* (*β*) errors, method used for estimating prevalence or standard deviation, percentage of meeting the expected sample size and considering clinically importance level in sample size calculation. Data were extracted from all included articles. Descriptive analyses were conducted. Inferential statistical analyses were done using independent T-test and Chi-square test with the significance level set at 0.05. **Results:** There was a statistically significant increase in years between 2009 and 10 compared to 2000-1 in terms of reporting sample size calculation *(P*=0.002), reporting clinically importance level (*P*=0.003) and in samples size of clinical trials (*P*=0.01). But there was not any significant difference between two journals in terms of reporting sample size calculation, type of control group, frequency of various study designs and frequency of positive and negative clinical trials in different time periods (*P*>0.05). **Conclusion:** Sample size calculation in endodontic clinical trials improved significantly in 2009-10 when compared to 2000-1; however further improvements would be desirable.

## Introduction

Evidence-based medicine (EBM) can be interpreted as the integration of the best and highest research evidence, clinical expertise and patient values, was first introduced by Dr. David Sackett in 1996 [1] and quickly gained dental practitioners’ attention in a short time. In EBM, double blinded randomized clinical trials are the gold standard for research methodology and provide valuable evidence in answer to clinical problems [2]. In clinical trials, the more accurate and unbiased the design of a study, the more reliable and relevant the results will be. 

The CONSORT guideline consists of a detailed checklist for reporting randomized controlled trials (RCTs), which is suggested for preparing the reports of RCTs According to this guideline, reporting the method of sample size calculation is mandatory for all clinical trials to be published [3].

Sample size calculation is essential before the start of RCTs to provide adequate power to detect significant differences among groups. If the sample size is not adequate, the likelihood of type *I* and *II* errors will increase. On the other hand, due to ethical and economic reasons, oversized trials need to be avoided. It is therefore important to optimize the sample size [4].

The importance of sample size calculation was emphasized in some published articles. In one study, 71 RCTs with negative results were reviewed to evaluate the sample size of each paper. Results showed that in most of the trials the power was not high enough to detect the differences [5]. In another study, the reporting of sample size calculation in RCTs published in 5 leading journals in the ﬁeld of physical medicine and rehabilitation (PM&R) was assessed systemically. They found that in 2008, 57.3% of the articles reported sample size calculation compared to only 3.4% in 1998 and that the articles often failed to report effect size for sample size calculation [6].

**Table 1:** Frequency of published clinical trials in two endodontic journals in 2000-1 and 2009-10

**Journal**	**Years**					
	**2000**	**2001**	**2009**	**2010**	**Total**
**International Endodontic Journal**	1	3	5	4	13
**Journal of Endodontics**	3	4	13	17	37
**Total **	4	7	18	21	50

**Table 2:** Sample size calculation criteria in two periods of time in two endodontic journals (*NS: non-significant*

	**Yes**	**No**	**Total**	***P*** **-value **	
**2000-1**	0 (0.0)	11 (100.0)	11 (100.0)	0.002	
**2009-10**	20 (51.3)	19 (48.7)	39 (100.0)		
**Total**	20 (40.0)	30 (60.0)	50 (100.0)		
**Method of estimation of prevalence or standard (%)**					
	**No sample size calculation**	**Previous studies**	**Pilot studies**	**Assumption**	**Total**
**2000-1**	11 (100.0)	0 (0.0)	0 (0.0)	0 (0.0)	11 (100.0)
**2009-10**	22 (56.4)	4 (10.2)	1 (2.5)	12 (30.0)	39 (100.0)
**Total**	33 (66.0)	4 (8.0)	1 (2.0)	12 (24.0)	50 (100.0)
**Reporting clinically importance level (%)**					
	**Yes **	**No**	**Total**	***P*** **-value **	
**2000-1**	0 (0.0)	11 (100.0)	11 (100.0)	0.003	
**2009-10**	19 (48.7)	20 (51.3)	39 (100.0)		
**Total**	19 (38.0)	31 (62.0)	50 (100.0)		
**Different types of control groups (%)**					
	**Alternative treatment**	**Placebo**	**Both of them**	**Total**	***P*** **-value **
**2000-1**	6 (54.5)	3 (27.3)	2 (18.2)	11 (100.0)	NS
**2009-10**	30 (76.9)	3 (7.7)	6 (15.4)	39 (100.0)	NS
**Total**	36 (72.0)	6 (12.0)	8 (16.0)	50 (100.0)	NS
**Different types of study design (%)**					
	**Parallel**	**Cross over**	**Total**	***P*** **-value **	
**2000-1**	11 (100.0)	0 (0.0)	11 (100.0)	NS	
**2009-10**	29 (74.4)	10 (25.6)	39 (100.0)	NS	
**Total**	40 (8.0)	10 (20.0)	50 (100.0)	NS	

An article by Greenstein *et al.* [7], compared clinical versus statistical significance in efficacy of periodontal therapy, and concluded that clinically important results should be defined before initiating a study and tests for determining the statistical significance should be used to validate those findings that did not occur by chance.

Usually, calculating the sample size for trials requires type *I* (*α*) and type *II* (*β*) error, event rate in the control group, and event rate in the treatment group. Also the smallest effect of interest as the minimal difference between the studied groups that the investigator wishes to detect, and the population variance of a given outcome variable, are needed [8].

Because of the importance of appropriate sample size in research methodology, exploring the sample size estimation in published high rank papers, might illustrate the weak points in this regard and help to generate more specific guidelines for writing scientific papers especially in the field of clinical trials.

**Table3:** Reporting sample size calculation in two endodontic journals (*NS: non-significant*

**Journal**	**Sample size calculation (%)**				
	**Yes**	**No**	**Total ** ***P*** **-value**	***P*** **-value**
**International Endodontic Journal**	3 (23.1)	10 (76.9)	13 (100.0)	NS
**Journal of Endodontics**	17 (45.9)	20 (54.1)	37 (100.0)	NS
**Total**	20 (40.0)	30 (60.0)	50 (100.0)	NS

The aim of this article was to evaluate the quality of reporting the sample size calculation in clinical trials published in two leading endodontic journals (*Journal of Endodontics* and *International Endodontic journal*) in years 2000-1 and 2009-10.

## Methods and Materials

The extracted data from each article consisted of the name of the journal, publication year, research design (parallel versus cross over), types of control group (alternative treatment, placebo or both), reporting sample size calculation (yes or no), the number of participants in each group, mean number of the participants in all research groups, study outcome (positive, negative), amounts of type I (α) and type II (β) errors, method used for estimating prevalence or standard deviation (previous studies, pilot studies or assumption), percentage of meeting expected sample size and considering clinically importance level in sample size calculation.

Data were extracted by two investigators (A.SH and M.R) and checked by other author (A.A.H). Any disagreements were resolved by a group consensus. Data were entered in Excel data sheet.

***Statistical analysis***

Statistical analysis was mainly done by descriptive methods. Also, comparing the means and proportions between groups was done by independent T-test and Chi-square analyses. A *P*-value of 0.05 was considered significant.

## Results

A total number of 50 clinical trials published in Journal of Endodontics and International Endodontic Journal in years 2000-1 and 2009-10, were reviewed. Frequency distribution of reviewed clinical trials in two journals in different years of publication can be seen in Table 1.

In 45 (90%) papers, type I error was 0.05 and the rest of the articles did not report any value for type I error. In 30 (60%) papers, Type II error was not reported, in 7 (14%) papers the value was 0.10, in 11 (22%) the value was 0.2 and in 2 (4%) papers, other values were reported for type II error.

A significant increase in terms of reporting sample size calculation in years between 2009 and 10 compared to 2000-1 was evident (*P*=0.002) (Table 2). But the difference between two journals regarding the report of sample size calculation was not significant (*P*>0.05) (Table 3). There was a significant increase in reporting clinically importance level in 2009-10 in comparison with 2000-1 (*P*=0.003) (Table 2). But the differences in type of control group in different periods of time and also the two periods of time in frequency of various study designs (Parallel or cross-over) was not significant (*P*>0.05) (Table 2).

There was a significant increase in sample size in 2009-10 (37.89±35.51) compared to 2000-1 (21.7±8.28) (*P*=0.01). Twenty five (50%) papers, had positive and 25 (50%) had negative results. There was not any significant difference for frequency of positive and negative clinical trials between two periods of time (*P*>0.05). Also there was no significant difference between positive and negative clinical trials regarding mean sample size per each group (*P*>0.05). The difference between the mean of sample size in clinical trials with parallel and cross-over designs, was not significantly different (*P*>0.05).

In all published papers which had reported the sample size calculation, authors met expected sample size which had been determined before beginning of the trials. Frequencies of positive and negative clinical trials between different types of control groups and also groups reporting or not reporting sample size calculation, did not show a significant difference, either (*P*>0.05).

## Discussion

None of the eleven clinical trials published in 2000-1 described sample size calculation, but in 2009-10, 51.3% of reviewed papers had considered reporting sample size calculation. Despite significant higher percentage of reporting sample size calculation in endodontic journals in recent years compared to 10 years earlier, in almost half of included papers, the method of sample size calculation was not reported. The rate of reporting sample size calculation in International Endodontic Journal and in Journal of Endodontics was 23.1% and 45.9%, respectively. In CONSORT guideline, the importance of describing sample size calculation is emphasized as an essential part in reporting clinical trials [3].

Of the total 50 published clinical trials in Journal of Endodontics and International Endodontic Journal in years 2000-1 and 2009-10, 37 papers were in Journal of Endodontics and 13 were in International Endodontic Journal. In recent years there has been a positive trend for publication of clinical trials in these journals. Considering increased attention to Evidence-based dentistry and the role of well-designed RCTs in answering clinical questions, this is not surprising. Abdul Latif *et al.* showed a 3-fold increase in publication of RCTs in 5 leading journals in physical medicine and rehabilitation from 1998 till 2008 [6], but Diener *et al.* showed that after 2000, there was declining frequency in publishing RCTs in German surgical journal Der Chirurg [9].

To identify all clinical trials published in endodontic journals, hand searching was done. Some authors mentioned that hand searching is still necessary and more sensitive in comparison to electronic searching alone to find published clinical trials [10].

Components of sample size calculation are type I (α) or type II (β) errors, effect size or clinical importance difference and variability or standard deviation in case of a continuous outcome. In this study, type I (α) and type II (β) errors were not reported in 10% and 60% of reviewed clinical trials, respectively. Type I error is often set at 0.05 but Type II error has been set at different values such as 0.10%, 20%, and etc. In another study which reviewed clinical trials published in journal of Physical Medicine and Rehabilitation, authors found that α and β parameters were set as 5% and 20%, respectively [6].

For sample size calculation in clinical trials, estimation of standard deviation or prevalence in both experimental and control groups, is essential. To have more accurate estimation, conducting pilot studies or extracting information from previous similar studies are recommended [8].

From 17 reviewed papers published between 2009 and 2010 in which the sample size calculation was reported, 4 papers used the previous studies for prior estimation of prevalence or standard deviation and in one paper, pilot study was used. In other 12 papers, the method of estimation was not mentioned.

Previous evaluations of RCT papers often showed signiﬁcant differences among groups, and conclusions were often based on this statistical signiﬁcance [11]. If *P-*value is less than 0.05, the difference is considered significant and if *P*-value is higher than 0.05, the difference between groups will be considered non-significant. This *P-*value is not very important and only shows what the probability of obtaining result only by chance, is. The *P*-value doesn’t indicate if the effect is clinically important. Clinically important results should be defined before initiating a study because it is possible for a procedure to provide a statistically significant improvement, while the result may not be clinically important. In 2000-1 none of the reviewed papers in this study reported clinically significant differences between groups but 48.7 % of reviewed papers in 2009-10, reported clinically important differences.

A study in Physical Medicine and Rehabilitation journal, which had evaluated sample size calculation authors showed that 26.8% of the trials reported a method for calculating effect estimation by clinically important effect size [6].

Another study compared statistically significant differences with clinical importance difference in clinical trials conducted on low back pain; of the 43 included studies, only six showed both clinically important and statistically signiﬁcant differences [11].

Researchers can consider control groups as active treatment (alternative treatment) or placebo or both. Seventy-two percent of reviewed articles in the current study had an active control group, 12% had placebo as control group and 16% had both. Clearly in endodontic clinical trials, researchers preferred an alternative treatment as a control group instead of placebo. Due to ethical consideration for using placebo as a control group in clinical research, these findings are sensible. This result is comparable to findings of a systematic review which evaluated the clinical trials in Physical Medicine and Rehabilitation journal. In that study, most control groups were active alternative treatments [6]. 

While none of reviewed clinical trials in 2000-1 had cross-over design, 20% of endodontic clinical trials in 2009-10, had cross-over design. The advantage of this design over a parallel group is that, the effects of the treatments are compared within the same patients. Cross-over design always requires fewer patients than the completely randomized ones [12]. In recent years, it is obvious that endodontic researchers use cross-over design to conduct clinical trials more than before.

Mean sample size per each group in clinical trials published in 2009-10 was 37.88 compared to 21.69 in 2000-1 and there was a statistically significant difference. Increasing sample size could be related to improved sample size calculation in endodontic clinical trials in recent years.

Number of positive and negative clinical trials was equal in reviewed articles (25 articles). Positive clinical trials were those studies with significant *P*-value for the difference in favor of the experimental group. In contrary, negative clinical trials, did not result in statistical significance. The mean sample size was not significantly different in positive and negative outcomes, and there was not any significant difference between proportions of result outcome in two-year groups. There was not a statistically significant difference in frequencies of positive and negative clinical trials between groups in which sample size calculation had been reported.

All of the reviewed clinical trials which had reported the sample size determination met expected sample size and there were more or at least equal participants in analysis portion of article compared to predetermined sample size.

## Conclusion

Reported sample size calculations in endodontic clinical trials has significantly improved in 2009-10 compared to 2000-1 but it still has room for improvement. The difference between “Journal of Endodontics” and “International Endodontic Journal” in reporting sample size calculation was not significant.
